# Antibody-labelled gold nanoparticles synthesized by laser ablation to detect SARS-CoV-2 antigen spike

**DOI:** 10.5599/admet.2079

**Published:** 2023-12-06

**Authors:** Asri Sulfianti, Vidhia Tiara Sopandi, Isnaeni Isnaeni, Jodi Suryanggono, Sabar Pambudi, Sjaikhurrizal el Muttaqien, Febby Nurdiya Ningsih, Tika Widayanti, Etik Mardliyati, Annisa Annisa

**Affiliations:** 1 Research Center for Vaccine and Drugs, National Research and Innovation Agency Republic of Indonesia (BRIN), LAPTIAB Building no 611-612, KST BJ Habibie, Serpong, Tangerang Selatan, Banten 15310, Indonesia; 2 Department of Biology, Faculty of Mathematics and Natural Sciences, Padjajaran University, Jalan Raya Bandung, Jatinangor, Sumedang, West Java 45361, Indonesia; 3 Research Center for Photonics, National Research and Innovation Agency Republic of Indonesia (BRIN), Physic Building no 15 442, KST BJ Habibie, Serpong, Tangerang Selatan, Banten 15314, Indonesia

**Keywords:** monoclonal antibody, anti-SARS-CoV-2, lateral flow immunoassay, Covid-19

## Abstract

**Background and purpose:**

Rapid detection test via lateral flow immunoassay (LFIA) is employed as an alternate method to detect Severe Acute Respiratory Syndrome Coronavirus 2 (SARS-CoV-2) infection. Gold nanoparticles (AuNPs), a vital component of LFIA, can be synthesized by laser ablation technique. This intense laser radiation may result in monodisperse gold nanoclusters, which are impurity-free and demonstrate innovative biocompatible surface chemistry. In this current research, laser-ablated AuNPs are produced and coupled with an anti-spike SARS-CoV-2 monoclonal antibody (mAb) generated in our prior study.

**Experimental approach:**

The AuNPs from 30,000 shots of laser ablation exhibited a robust red color with a maximum absorbance peak at 520 nm. The performance of AuNPs-mAb conjugates as a signal reporter was then evaluated in half-stick LFIA.

**Key results:**

The size distribution of AuNPs shows a relatively monodisperse and unimodal distribution with average particle diameters of 44.77 nm and a surface potential of -38.5 mV. The purified anti-spike mAb SARS-CoV-2 yielded two protein bands, representing the mAb heavy chain at 55 kDa and its light chain at 25 kDa. The immobilization of anti-spike mAb onto the surface of AuNPs revealed that 25 g/ml of mAb at phosphate buffer pH 9 was required to stabilize the AuNPs. The functional test of this conjugate was performed using dipstick LFIA, and the result shows that the AuNPs-mAb conjugates could successfully detect commercial spike antigen of SARS-CoV-2 at 10 ng level.

**Conclusion:**

In this study, laser-ablated AuNPs were functionalized with anti-spike mAb SARS-CoV-2 and successfully used as a signal reporter in half-stick LFIA for detecting antigen spike SARS-CoV-2.

## Introduction

Since late 2019, Coronavirus Disease 2019 (COVID-19) has been spreading rapidly in many countries and has become a global epidemic. This disease is caused by the Severe Acute Respiratory Syndrome Coronavirus 2 (SARS-CoV-2), a positive-sense, single-stranded enveloped RNA virus from the Coronaviridae family with a genomic length of more than 30 kb. The 5' terminus of the SARS-CoV-2 genome encodes 16 non-structural proteins (NSP1-16), which will form the replicase/transcriptase complex (RTC). In comparison, the 3' terminus will encode four main structural proteins: spike (S), envelope (E), membrane (M), and nucleocapsid (N). According to World Health Organization (WHO), this virus infection in humans can range from asymptomatic to mild sickness, with disease mortality rates higher among the elderly with chronic comorbidities [1-4].

In recent literature, various approaches for particular virus detection have been developed, and all of them are based on detecting viral nucleic acids during acute infection. With this knowledge, the reverse transcription-polymerase chain reaction (RT-PCR) has been considered the gold standard for diagnosing COVID-19 [5]. However, the RT-PCR method possesses some limitations, including lengthy ribonucleic acid (RNA) extraction steps and the inability to do on-site detection. Furthermore, this technique requires highly skilled operators and complex bio-safety equipment. To address these difficulties and the growing demand for rapid diagnostic tests and a regionally decentralized healthcare system, point-of-care testing (POCT) kits in remote areas have grown exponentially [2-6].

On the other hand, biosensors have gained popularity as simple tools for disease diagnosis. Biosensors are classified into several categories, including chip-based, enzyme-based, immuno-sensors, and Deoxyribonucleic acid (DNA) sensors [7,8]. Lateral-flow immunoassay (LFIA) is a biosensor that is being developed for biological testing and has emerged as one of the most appealing POCT applications. Because of its ease of use and quick findings, LFIA is currently commonly utilized for screening COVID-19. Furthermore, the lateral flow test strip is the largest platform segment of the POC diagnostic market, owing to LFIA's superiority over lengthy, conventional laboratory procedures or other platforms such as electrochemical biosensors that require specific devices, a large sample volume, and time-intensive analysis. In addition, it is challenging to develop electrochemical sensors for commercial personal computer (PC) devices used in clinical applications [2,6,9,10].

Theoretically, in the LFIA, the detection works through the movement of the fluid sample on the polymeric membranes by the capillary action. It successively interacts with various reagents to produce a detection signal [11-13]. Gold nanoparticles (AuNPs) are one of those materials that can be used as signal labels in various sensor platforms. Due to their biocompatibility and specific optical characteristics, AuNPs are commonly used in constructing LFIAs [14,15]. The interaction of AuNPs with analytes, such as proteins and antibodies, changes the physicochemical properties of AuNPs, which in turn generate observable signals for target identification and quantification [16,17].

Nowadays, AuNPs are synthesized mainly by chemical reduction of the gold precursor tetra chloroauric acid (HAuCl_4_). This traditional technique, however, requires an extra purification step to remove surface contamination, such as residual anions, ligands, and reducing agents from the colloidal suspension [18]. In long-term usage, this procedure also produces toxicity, which complicates its in-vivo applications. Furthermore, no universal reducing agents have been identified to produce monodisperse AuNPs [19].

In recent years, laser ablation of gold (Au) in aqueous media has been used to create AuNPs. The Au target immersed in aqueous media is ablated by solid laser radiation, resulting in Au nanoclusters. These nanoclusters then combine to make a stable nanoparticle (NPs) dispersion. These laser-synthesized AuNPs are impurity-free because no foreign ligands are on the surface. Thus, it will save manufacturing costs [20]. Furthermore, laser-synthesized NP can exhibit distinct surface chemistry, making them reactive with a wide range of novel biocompatible materials. For instance, because of the occurrence of some higher oxidation states at high pH, they can have O functionalization, allowing for hydrogen bonding interactions. Therefore, non-covalent conjugation of Au-NPs to oligosaccharides, polymers, proteins, and oligonucleotides is possible. Such direct conjugation (without any group ligand-specific binding) promotes Au-NPs as novel classes of biocompatible materials for various underdeveloped or unexplored biological applications [21].

In this present study, we develop direct detection of SARS-Cov-2 antigens to confirm the presence of the virus. The AuNPs synthesized by laser ablation technique will be conjugated with anti-spike SARS-Cov-2 monoclonal antibody (mAb) to detect the spike protein. Spike glycoprotein and N phosphoprotein of SARS-Cov-2 are the most viral structures exploited in early diagnosis and detection of circulating viruses in the organism. Moreover, biosensors targeting the spike protein in relevant body fluids allow for detecting entire virus particles [3,5]. The AuNPs are generated by laser ablation at 30.000 shots, and anti-spike SARS-Cov-2 mAb is produced *in vitro*. A physical absorption technique is used to conjugate anti-spike mAb and AuNPs non-covalently. The functional test of this conjugate as a signal reporter is performed in half-stick LFIA using a nitrocellulose membrane (NC) and an absorbent pad as the LFIA platform. Instead of a line test, the dots, as in the dot blot test, are used in this test assay model [22].

## Experimental

### Synthesize and characterization of gold nanoparticles (AuNPs)

**Figure 1.**
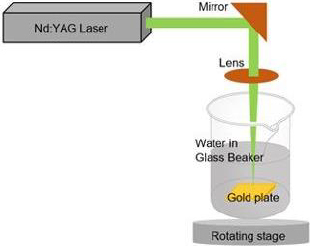
Schematic procedure of gold nanoparticles synthesis

Laser ablation was used to create AuNPs in an aqueous medium. The gold plate 99.99 % from PT. Aneka Tambang (ANTAM) Tbk. was placed at the bottom of the deionized water-filled glass beaker, as shown in Figure 1. The gold plate was cleaned using ethanol prior to the experiment. A nanosecond pulsed neodymium-doped yttrium aluminium garnet (Nd: YAG) laser with excitation wavelength 1064 nm and power of 60 mJ was focussed on the surface of the gold plate using a 150 mm focusing lens. The gold plate was shifted across the focusing plane at a constant speed of 0.5 mm s^-1^ while keeping the same thickness of the liquid above the object of around 1 cm. The process was conducted for 30.000 shots to ablate the gold plate until the liquid turned red. The synthesized gold nanoparticles were then used in the experiment without further purification. The absorbance of the AuNPs was measured at numerous wavelengths using ultraviolet (UV) spectrometry to determine its maximum wavelength. The size of AuNPs was determined using the dynamic light scattering (DLS) technique, and the surface charge of AuNPs was observed using a zeta-sizer. In addition, the size of synthesized gold nanoparticles was observed by transmission electron microscope (TEM) [23].

### Anti-spike SARS-CoV-2 monoclonal antibody production

In this study, the hybridoma cell clone, which produces anti-spike SARS-CoV-2, was previously created by the monoclonal antibody research group at the Research Center for Vaccine and Drugs, BRIN. All procedure to generate the hybridoma cell was approved by the Health Research Ethics Committee for Animal Experiments, Faculty of Medicine, University of Indonesia (No. 375/UN2.F1/ETIK/PPM.00.02/2021) with protocol number 21-02-0197. A hybridoma cell clone SF2 was cultured in 75 T-Flasks with 10 ml of Roswell Park Memorial Institute (RPMI) media supplemented with 3 % Foetal Bovine Serum (FBS) and 1 % Penicillin-Streptomycin. The cells were then incubated at 37 °C in a 5 % CO2 enriched until they reached 80 % confluence. The cells were then passaged, and the antibody was produced in a serum-free medium (SFM). Cells (2x10^4^ cells ml^-1^) were cultivated in 300 ml of SFM in 500 ml of stirred-Duran bottle. The cell was then incubated at 37 °C for five days to evaluate the growth curve and cell growth rate to obtain maximum production time.

### Purification of anti-spike SARS-CoV-2 monoclonal antibody

On the maximum antibody production day, the culture supernatant was harvested by centrifuge at 10.000 rpm for 30 min at 4 °C. Subsequently, the supernatant was concentrated using Amicon® Ultra-4 10K device (10.000 MWCO) ultrafiltration centrifuged at 3.500 G for 30 minutes at room temperature. The concentrated product was filtered via a 0.45 m syringe filter. Furthermore, before loading into the purification column, the concentrated product was diluted 1:1 with a binding buffer (0.02 M Na-phosphate buffer pH 7.4) and centrifuged at 10.000 g for 15 minutes. A purification column was prepared by streaming 2 ml of resin G in 20 % ethanol with 10 column volumes of binding buffer. The obtained concentrated product (100 ml) was gravity-passed over the column at least twice. The column was then thoroughly cleaned until no protein was identified in the eluent. Additionally, elute bound antibodies with 0.1 M glycine (pH 2) and collect fractions in tubes containing 2 M Tris-Cl (pH 8). Furthermore, the antibody buffer was replaced with Na-phosphate using an Amicon column.

### Quantifying the concentration of purified anti-spike SARS-CoV-2 monoclonal antibody

Pierce bicinchoninic acid (BCA) protein assay determined the total protein concentration. The technique starts with diluting the bovine serum albumin (BSA) into nine concentrations (0 to 2.000 μg ml^-1^). Each tube received 25 μl of each standard and protein sample replicated and mixed with 200 μl of the working reagent buffer. After microtubes were incubated in dark conditions at 37 °C for 30 minutes, they were cooled to room temperature, and the absorbance of the standard and samples was measured at 562 nm. The protein concentration was calculated using linear regression to the standard curve.

### Characterization of anti-spike SARS-CoV-2 monoclonal antibody

The purified antibody was visualized using sodium dodecyl sulphate–polyacrylamide gel electrophoresis (SDS-PAGE) with a 12 % acrylamide gel. In addition, an in-house enzyme-linked immunosorbent assay (ELISA) was used to test the functionality of the antibody against inactivated SARS-CoV-2 viruses (Sinovac-CoronaVac COVID-19 vaccine). In each 96-well plate, 50 ng ml^-1^ Sinovac was diluted with 100 μl of coating buffer, except for the negative control, which only received a coating buffer. The plate was incubated at 37 °C for 2 hours. The coating buffer was removed from the well plate and rinsed five times with PBST 0.1 %. The plate was blocked by adding 100 μl of 5 % BSA in phosphate buffer saline-tween (PBST) 0.1 % and incubated overnight at 4 °C. The blocking buffer was removed from the well plate and rinsed five times with PBST 0.1 %. 50 μl of pure anti-SARS-CoV-2 spike were put into the well in triplicate. 50 μl of SFM media triplicate was added for the negative control, whereas 100 μl of commercial anti-spike SARS-CoV-2 (1:1000) was added to the positive control well.

Furthermore, the plate was incubated at 4 °C overnight. After that, the entire solution was discarded and rinsed five times with PBST 0.1 %. 100 μl of secondary antibody (stabilized peroxide conjugated-goat anti-mouse (1:5.000)) was applied into the wells and incubated for one hour on a rotating shaker at 37 °C. Hereafter, all secondary antibodies were removed from the well plate and washed five times with PBST 0.05 %. 100 μl of 3,3′-5,5′ tetramethylbenzidine (TMB) was added to the well and incubated at room temperature in the dark condition until the color changed to yellow. 50 μl of 1 M sulfuric acid (H_2_SO_4_) was then added to the well as a stop solution, and the color changed from yellow to blue. The absorbance of the solution was then measured at 450 nm in an ELISA reader.

### Adjusting optimal pH for AuNPs conjugation

Before each experiment, each of 100 μl of AuNPs solution was adjusted to the desired pH (6,7, 8, and 9) with phosphate buffer 0.02 M. 0.5 μg ml^-1^ of purified anti-spike SARS-CoV-2 mAb was added to each microtube and incubated at rotator at 80 rpm for 30 minutes. Sodium chloride (NaCl) solution (10 %, 2 μl) was added into each microtube, and the AuNPs colors were observed, followed by the absorbance measurement at wavelengths of 500 to 600 nm. The optimum pH is indicated by no color change of AuNPs dispersion. The highest absorbance value of the AuNPs was chosen for further conjugation procedure.

### Estimation of AuNPs and antibody conjugates

A series of purified anti-spike SARS-CoV-2 monoclonal antibodies were prepared at a range of 1.25 to 50 μg ml^-1^ in phosphate buffer. To 100 μl of each laser-ablated AuNPs and commercial AuNPs, 10 μl of antibodies were added. After 30 minutes of stirring at 80 rpm on a rotary shaker, 10 % NaCl was added to each antibody concentration. The absorbance of solutions was then measured at 500-600 nm. Flocculation curves were drawn as a function of absorbance at a maximum wavelength in the existence of 10 % NaCl. The optimal amount of antibody was obtained when the mixture could maintain the original red wine color of AuNPs after adding 10 % NaCl.

### Synthesize AuNPs-antibody conjugates

Anti-spike SARS-CoV-2 monoclonal antibody at optimum concentration was combined with 100 μl of laser-ablated AuNPs and commercial AuNPs (Cyto-diagnostic Cat. G-40-XX) at the optimum pH. The mixture was stirred at 80 rpm for 30 minutes before being centrifuged at 10.000 rpm for 10 minutes to separate unbound antibodies. Then, a final concentration of 5 % BSA was added and incubated as before. The mixture was centrifuged at 10.000 rpm for 10 minutes to separate the unbound BSA. After removing the supernatant, 20 μl of precipitate was resuspended in a phosphate buffer containing 1 % BSA. The conjugate's absorbance was determined using a spectrophotometer at 500 to 700 nm wavelength.

### Determination of antigen-capturing activity of the conjugate

Half-stick LFIA was used to test the antigen-capturing activity of the mAb-AuNPs conjugate. LFIA nitrocellulose membrane (NC, 8 μm) was trimmed to 0.5 cm and connected to the absorbent pad. The NC membrane was immobilized with 1 μl of commercial capture anti-spike SARS-CoV-2 at 1 mg ml^-1^ as a test dot and 1 μl of anti-mouse IgG polyclonal antibody at 1 mg ml^-1^ as a control dot. The NC membrane was then dried for 1 hour at 37 °C. The commercial spike antigen (10 ng) was mixed with 20 μl of mAb-laser-ablated AuNPs conjugate. After drying the NC membrane, it is dipped into the 96-well plate previously filled with an antigen-conjugate mixture. As it moved with capillary forces to the NC membrane, 30 μl of BSA 5 % at PBST 0.1 % was poured into the well. After 10 minutes of incubation, the NC membrane was washed with the buffer to remove the red background stain. The color intensity of the control and test dots and the presence and absence of red dots at the test zone were evaluated for each set. In comparison, the functional test was also conducted by using mAb-commercial AuNPs conjugate.

## Results and discussion

In diagnostic tests, susceptible regions on viral surface proteins are widely used as an antibody’s primary target. In this study, the conjugation of anti-spike mAb and ablated AuNPs was developed to detect the spike protein of the SARS-CoV-2 virus. Coronavirus-neutralizing antibodies predominantly target the viral surface's trimeric spike glycoproteins, which mediate the entry of pathogens into host cells. This protein has two functional components that facilitate cell attachment (the S1 subunit, which comprises the N-terminal domain (NTD) and receptor-binding domain (RBD)) and viral-cellular membrane fusion (the S2 subunit) [3,4,23,24]. Because the recombinant SARS-CoV-2 S protein has become widely accessible just a few months after the outbreak, a more straightforward and quicker ELISA based on Angiotensin-converting enzyme 2 - receptor-binding domain (ACE2-RBD) interaction may be quickly developed for detecting SARS-CoV-2. Lateral flow approaches for detecting SARS-CoV-2 were developed by utilizing a similar technique and the same set of recombinant proteins as ELISA [25]. In this study, we developed a half-stick lateral flow immunoassay for commercial spike antigen detection employing anti-spike mAb conjugated with ablated AuNPs.

### Synthesize and characterization of AuNPs

Firstly, AuNPs were synthesized by laser ablation technique with 30.000 shots. Theoretically, when AuNPs are synthesized, they appear in an aqueous solution in a variety of colors (*e.g.*, brown, orange, red, and purple) as the core size increases from 1 to 100 nm and absorption peak spans from 500 to 550 nm [11,26]. This absorption band, called the surface plasmon band, comes from the collective oscillation of conduction electrons caused by resonant stimulation by input photons. However, this band is missing in both small nanoparticles (*d* = 2 nm) and bulk material. This phenomenon was affected by shape, solvent, surface ligand, core charge, temperature, and proximity to other nanoparticles. The NPs aggregation generates a substantial red shift in surface plasmon resonance (SPR) frequency, a broadening of the surface plasmon band, and a change in solution color from red to blue due to interparticle plasmon interaction [11,16,26].

**Figure 2.**
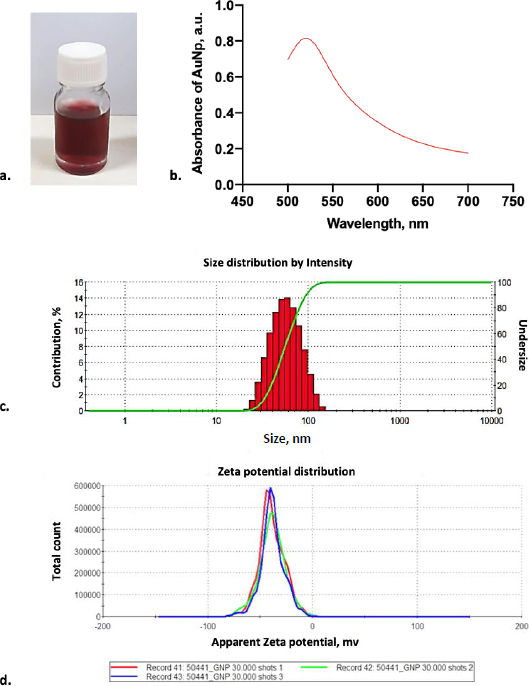
Characterization of gold nanoparticles (AuNPs). (a) Colloidal suspense of AuNPs; (b) AuNPs' maximum absorption at 520 nm; (c) The average diameter of AuNPs was seen at 44.77 ± 0.264 nm; (d) The surface charged by the zeta sizer indicates the AuNPs were negatively charged with an average surface at −38.5 ± 12.1 mV

As shown in Figure 2a, the laser ablation process in deionized water solution resulted in a transparent red color dispersion. A high concentration of gold nanoparticles was achieved using the laser ablation technique. The colloidal gold nanoparticle solution was stable for several days without forming aggregation. After over a week, the gold nanoparticles started to agglomerate to form sedimentation in the bottom of the bottle. Based on this observation, the newly fresh gold nanoparticles were used during the conjugation experiments. In order to check the reproducibility of gold nanoparticles, the absorbance spectra of gold nanoparticles were measured. From absorbance spectra, the AuNPs produced by laser ablation had a deep visible red color in milli-Q water, with a prominent absorbance peak at 520 nm (Figure 2b) and an absorbance value of 0.848. The narrow peak shape of the UV-Vis absorption spectra suggests that these AuNPs have homogenous dispersion [15].

In order to clarify the shape and size distribution, we measured the produced gold nanoparticles using DLS and TEM. The principle of DLS involves measuring scattered light from a laser passing through the colloid. The intensity of scatter light modulation is analyzed throughout time to estimate the size of hydrodynamic particles [26]. According to the DLS result, monodisperse AuNPs were observed with an average diameter of 44.77 ± 0.264 nm (Figure 2c). Furthermore, electrostatic and hydrophobic interactions between the antibody and the gold surface resulted in non-covalent binding forms. The attraction between the hydrophobic region of the antibody and the metal surface results in the creation of non-covalent bonds during hydrophobic interactions. Positively charged amino acids, such as Lysine groups, which are prevalent in antibodies, generated electrostatic interactions with the negative charge of a carboxylated AuNP’s surface [15,27,28]. Based on our result, identifying the AuNP surface charged by the zeta sizer showed that it was negatively charged with the average surface of -38.5 ± 12.1 mV (Figure 2d).

**Figure 3.**
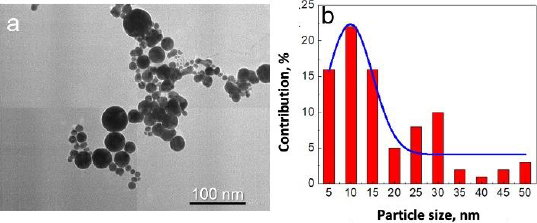
(a) TEM image of gold nanoparticles synthesized using laser ablation technique and (b) size distribution of gold nanoparticles

In addition, the morphology of gold nanoparticles was observed by TEM. Although the images rendered by TEM are two-dimensional, this system can deliver a much greater solution [26]. TEM visualization on AuNPs showed that the shape of gold nanoparticles was spherical for particles larger than 20 nm in diameter and almost spherical for smaller gold nanoparticles, as shown in Figure 3a. Most of the gold nanoparticles are monodispersed. Only a small part of the gold nanoparticles was agglomerated. The agglomeration occurred due to the absence of any passivation during the synthesis. Most chemically synthesized gold nanoparticles used passivating agents such as polyethylene glycol (PEG) and citrate [26,27].

Based on observation, the synthesized gold nanoparticles were of very high purity since no additional chemicals were used in the synthesis procedure. The advantages of these fabricated gold nanoparticles were the high purity and simple fabrication of gold nanoparticles [20]. However, although most of the gold nanoparticles were seen between 5-15 nm in diameter, some particles were more prominent than 25 nm in diameter (Figure 3b). Fortunately, for the next conjugation step, the size deviation of gold nanoparticles did not significantly affect the results.

### Anti-spike SARS-CoV-2 monoclonal antibody production

**Figure 4.**
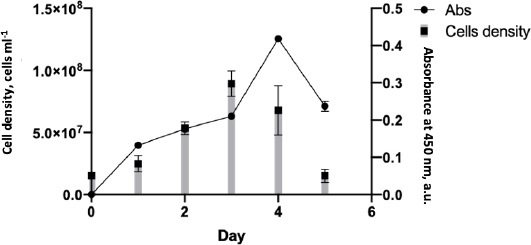
Growth curve of SF2 hybridoma clone cells secreting anti-SARS-CoV-2 monoclonal antibody

During the mAb production, the cell growth rate was monitored to determine the maximum time for hybridoma cells to produce an anti-SARS-CoV-2 spike. The cell growth rate formula [14] calculated that the cell growth rate from days 1-3 was 0.024, with the highest number of mass cells on day 3. After that, the cell growth rate decreased by -0,037 points on days 4-5, eventually returning to the initial level of 15 x 10^6^ (Figure 4). Although cell mass was abundant on day 3, the highest antibody detected by the ELISA test was on day 4, with an absorbance of 0.4.

According to the antibody production, the hybridoma SF2 secreting anti-spike SARS-CoV-2 2 could grow on media without serum supplementation. The use of serum in biological products has many concerns regarding its scientific, technical, financial, and ethical issues [29]. The culture supernatant was collected and purified on day 4, the day of greatest antibody production.

### Purification of anti-SARS-CoV-2 spike monoclonal antibody

**Figure 5.**
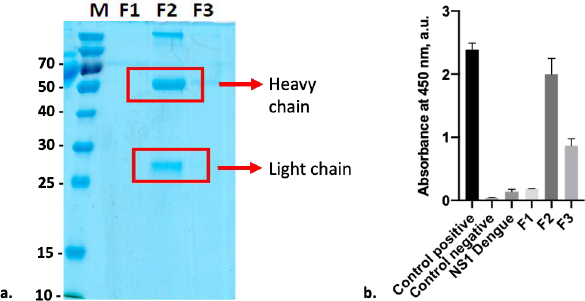
Characterization of purified anti-SARS-CoV-2 spike monoclonal antibody in SDS-PAGE and its functional test with ELISA assay: (a) Two distinct protein bands at 55 and 25 kDa represent heavy and light chains of anti-SARS-CoV-2 spike antibody, respectively. M: Ladder protein marker; F1-3: fraction 1-3; (b) ELISA assay performance to purified anti-SARS-CoV-2 spike antibody

Anti-SARS-CoV-2 spike monoclonal antibody was purified using resin G, resulting in two distinct protein bands that can be seen at Fraction 2 (F2). Both bands were at 55kDa and 25kDa, representing the heavy and light chains, respectively (Figure 5a). Based on the observation, SDS-PAGE did not reveal any protein band in F1 and F3; however, anti-spike captured with ELISA to all fractions revealed that these fractions contained antibodies. According to the data, F2 exhibited the highest antibody absorbance against the Sinovac vaccine, followed by F3 and F1 (Figure 5b). This finding is consistent with immunoglobulin (Ig) comprising four polypeptide chains. Each polypeptide has two identical heavy chains (HC) with a molecular weight of 53 to 75 kDa and two identical light chains (LC) with a molecular weight of 25 kDa [30]. On the other hand, the detection of the SINOVAC vaccine by commercial anti-S revealed that the level of detection of antigen on F2 was as high as its positive control. Furthermore, ELISA cross-reactivity with the Dengue non-structural 1 (NS1) product showed that F2 had no cross-neutralizing activity with the Dengue non-structural protein. A further examination with BCA assay shows that the protein concentration of F2 was 292 μg ml^-1^.

### Characterization of AuNPs-anti-spike SARS-Cov-2 monoclonal antibody conjugates

The AuNp-antibody conjugate binds to the biomarker of interest in dipstick sandwich immunoassay. This results in the visible test line color, the ultimate readout to deliver the diagnosis. Many studies have demonstrated that the interface between protein and AuNP can significantly impact the protein's structure and function. This phenomenon is due to the surface effects of the protein interacting with the AuNp surface or surface coating ligand. In our study, the conjugation of AuNp-antibody was carried out by adsorption [27].

AuNPs are typically very sensitive to salt concentration. As the cations are present in salt solutions, this charge repulsion is neutralized and causes the agglomeration of the AuNPs. Thus, adding aqueous NaCl to typically stabilized particles covered the surface charge, lowering the interparticle distance and finally producing particle aggregation [20]. As a result, protein binding to AuNPs would maintain the suspended particles by preventing salt-induced precipitation of the colloidal AuNPs. Thus, it is critical to find the optimized antibody concentration and pH during antibody conjugation for obtaining high antigen-capturing activity. The optimum antibody is required to produce a stable conjugation that could cover the whole surface of the AuNPs. The flocculation curves for the AuNPs were used to determine the stabilizing concentration of the mAb. The addition of 10 % NaCl solution modifies the electric double layer of AuNPs and shifts the balance between electrostatic repulsion and attraction force, resulting in the flocculation of the AuNPs [20,31].

**Figure 6.**
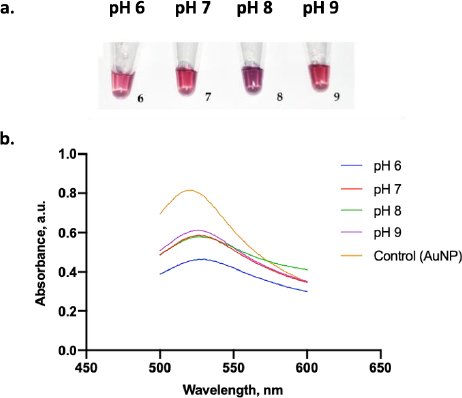
Adjusting optimal pH for AuNPs conjugation: (a) Photographs of several AuNPs incubated with 0.5 μg/ml of pure anti-spike SARS-CoV-2 monoclonal antibody at various pH and (b) its spectral analysis

Figure 6a displays the color change of the AuNPs under various pH conditions. A clear red color was visually observed when AuNP dispersion was adjusted to pH 6, 7, and 9, while those at pH 8 displayed a purple color. From the absorbance test, only AuNPs at pH 9 had the highest absorbance value compared to the other pH (Figure 6b). Thus, pH 9 was the optimum pH to stabilize the AuNPs. Moreover, adding 10 % NaCl to AuNPs below pH 9 induced a particle agglomeration and led to a color change of the dispersion from red to purple [32].

Besides the pH effects, temperature is another parameter impacting AuNPs' stability. Several experiments found that the reaction also increases as the temperature rises. This phenomenon is because an increase in temperature can speed up the reaction by providing more thermal energy, thus increasing the kinetic energy of the molecules [33]. The study by Sun *et al.* [34] demonstrated that alterations in pH and temperature caused shifts in the "steric covering" of the protein's active site, achieved through interpolymer hydrogen bonding.

Additionally, according to Saira *et al.* [35], the stability of AuNPs at different temperatures was examined. AuNPs of a specific size remained stable within a range of temperatures, enduring collisions of reactants and solvent molecules without dissolving their surface atoms. However, as the temperature increased from 45 to 50 °C, signs of catalyst dissolution began to be found, evidenced by a gradual decrease in optical density (OD) values. This dissolution was attributed to the higher kinetic energy of molecules at elevated temperatures, leading to the surface atoms dissolving into the reaction mixture. This process continued with further temperature increases (50 to 60 °C), but no aggregation was observed [35].

In our investigation, however, we used a 10 Hz pulse laser with a 100 ms delay between pulses. This time is sufficient to stabilize the plate temperature and laser focus position. This finding is in line with Kim et al. [36], which found that the agglomeration event does not occur within milliseconds because the pulse width is under five ns. Thus, even if agglomeration occurs, the chance is relatively low because it occurs during those five ns [36].

**Figure 7.**
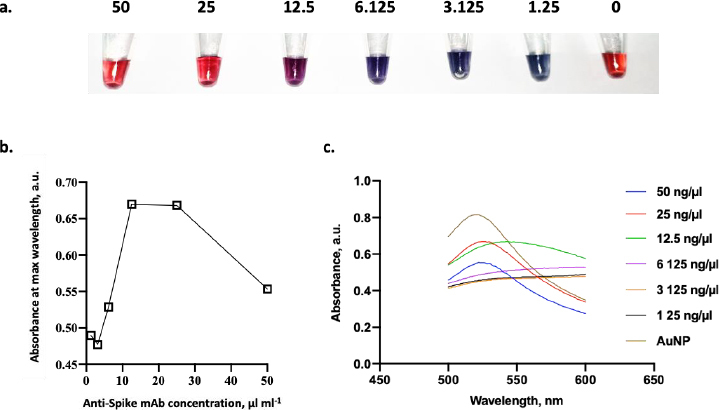
(a) Photograph of different aliquots of AuNPs supplemented with different concentrations of antibodies (1.25 to 50 μg ml^-1^ ); (b) Flocculation curve of antibodies immobilization to the AuNPs surface; (c) Spectral analysis of AuNPs incubated with various concentrations of antibodies

In addition to the environmental conditions, it is essential to determine the optimal concentration of conjugated mAb in this study. In this experiment, several concentrations of anti-spike mAb, ranging from 1.25 to 50 μg ml^-1^, were incubated with AuNPs suspension. Figure 7a shows the color change of AuNPs containing anti-spike mAb coupled AuNPs in various ratios. The color of the dispersed AuNPs suspension changed to blue after a small number of antibodies were added in the presence of NaCl. In contrast, when the optimum amount of mAb was added, the stable AuNPs could be achieved, and the suspension remained stable even after the addition of NaCl [20]. According to the result, the flocculation was seen from 1.25 to 12.5 μg ml^-1^ of anti-spike mAb concentration, and it was shown that 25 μg ml^-1^ of anti-spike mAb could stabilize the AuNPs in pH 9. Conjugates generated at antibody concentrations lower than the flocculation limit were not stable with the addition of salt, as seen by the color shifting from red wine to blue.

As shown in Figure 7b, the minimum amount of anti-spike mAb needed to stabilize the conjugate was 25 μg ml^-1^. Besides that concentration, the conjugate agglomerated as indicated by the color changing from red to purple in the presence of 10 % NaCl. Furthermore, antibody binding to colloidal AuNPs displayed saturation kinetics as there is a limited binding site of mAb on the surface of AuNPs [20]. Then, the colloid absorption was measured to assess the stability of AuNPs after adding mAb. The adsorption peak of stabilized AuNPs with anti-spike mAb is red-shifted by several nanometres to 520 nm at a concentration of 25 μg ml^-1^ of antibody (Figure 7c). In comparison, those without any mAb were 518 nm. In addition, the comparison between UV-Vis spectra before and after conjugation validated the conjugation’s success. The peak of UV-vis spectra following conjugation was red-shifted from 519 to 524 nm, confirming the enlargement of the gold nanoparticle size from the attachment of the antibodies to the nanoparticle [37].

### Determination of antigen-capturing activity of the conjugate

A commercial anti-spike monoclonal antibody was used to validate the reaction, and an anti-mouse IgG non-HRP polyclonal antibody was immobilized on the NC membrane in a half-stick dot blot assay. The sandwich format will display the competitive reaction between commercial anti-spike and AuNPs labelled anti-spike monoclonal antibodies in capturing different antigen epitopes in a sample. The reaction will appear as a red spot in a test dot as AuNPs labelled antibodies generate the red signal. In addition, another red spot will also appear in the control dot as anti-mouse IgG directly captured AuNPs labelled anti-spike monoclonal antibody either with the presence or absence of antigen in the sample as a control system. This finding ensures that the conjugated antibody works appropriately and that the sample has reached the end of the LFIA strip via capillary action [38].

**Figure 8.**
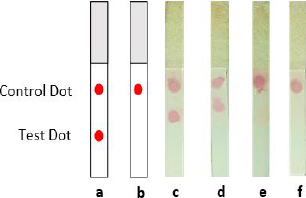
Functional test of conjugated anti-spike and laser ablated- gold nanoparticle by using half-stick LFIA platform: (a) Illustration of a positive result; (b) Illustration of a negative result; (c) Detection spike antigen with mAb- laser-ablated AuNPs conjugate; (d) Detection spike antigen with mAb-commercial AuNPs conjugate; (e) Antagonistic control mAb- laser-ablated AuNPs conjugate; (f) Negative control mAb-commercial AuNPs conjugate.

Four distinct NC membranes with varying treatments are displayed in Figure 8. The test and control dots were represented by two red blots on the NC membrane that were spotted with mAb and anti-mouse IgG antibodies (Figure 8a). In contrast, the negative control sample (Figure 8b) showed only one red point of the control dot. According to this assay, the antigen of the spike could be detected by both mAb-AuNPs conjugates (laser-ablated and commercial AuNPs) at a concentration of 10 ng (Figure 8c-d), whereas the negative control sample (Figure 8e-f) showed just one red spot. All test strips in this assay produced a detection signal within 10-15 minutes. In comparison, another experiment found that the limit of detection for LFIA spike targeting was varied, as the LOD was 25 ng *per* test. Additional optimization parameters are required, including determining the optimal antigen extraction reagent, buffer, and pre-incubation times [39].

The accumulation of AuNPs-tagged antibodies at the test and control zones resulted in the formation of visible red dots. The highly red spots developed due to surface electron collective oscillations triggered by visible light of an appropriate wavelength [38]. Thus, the appearance of a test and control dot revealed the presence of spike SARS-CoV-2 in the sample (positive result). If only a control dot appeared, the spike is absent in the sample (negative result) [40]. However, the result was deemed invalid if no red line emerged at the control line. Finally, Tween-20 was added in the PBST 0.1 % chase buffer as a detergent in the washing stage since the background residue might comprise both soluble and insoluble matrix [9].

Based on these findings, there is potential to advance the LFA technique as a prototype for rapid detection of SARS-CoV-2 and even other diseases. However, it is crucial to understand the expiration of this prototype, which currently, our research has yet to have its data due to the necessity for prolonged observation. Based on Parolo *et al.* [41], certain surfactants utilized in LFIA strips to enhance paper flow or label particle release can potentially diminish protein longevity. Furthermore, the membrane has an expiration date, evidenced by yellowing, odor, and potential faint lines post-assay, indicating deteriorated nitrocellulose. To extend nitrocellulose lifespan, it should be stored in dry conditions shielded from direct light, such as in a sealed bag with desiccants [41].

Interestingly, producing gold nanoparticles using laser ablation is relatively cost-effective due to the minimal amount of about 0.4 mg of gold required to generate 20 ppm of nanoparticles in 20 ml of liquid water. Creating AuNP nanoparticles by laser ablation was extremely promising. Rahmadianti *et al.* [42] discovered that creating AuNP nanoparticles by laser ablation was extremely promising, the total cost of producing 100 g of AuNP nanoparticles daily is $ 2,323,180 annually. According to the payback period (PBP) study, the investment will be worthwhile after more than 2.2 years. Because of the short investment returns, this project can compete with PBP capital market criteria. To assure project feasibility, the project is estimated from ideal to worst-case production settings, including labor, sales, raw materials, utilities, and external variables [42].

## Conclusion

In this study, laser-ablated AuNPs produced monodisperse AuNPs with an average diameter of 44.77 nm. The morphology of gold nanoparticles revealed that the shape was spherical with high purity because no additional chemicals were used in manufacturing. Anti-SARS-CoV-2 spike monoclonal antibodies, on the other hand, produced two different protein bands (55 kDa and 25 kDa), representing the heavy and light chains, respectively. According to the laser-ablated AuNPs-antibody conjugation, 25 μg ml^-1^ of anti-spike mAb could stabilize the AuNPs at pH 9. The sandwich format will demonstrate the competitive interaction between commercial anti-spike and AuNPs labelled anti-spike monoclonal antibodies to confirm the functionality of this conjugation. Based on this experiment, both mAb-AuNP conjugates (laser-ablated and commercial AuNPs) detected spike antigen at a 10 ng concentration. Two red blots on the NC membrane spotted with mAb and anti-mouse IgG antibody, representing test and control dots, were seen. In contrast, only one red control dot spot was seen in the negative control sample. Within 10-15 minutes, all test strips in this assay produced a detection signal.

## Future perspective

The prospects of lateral flow immunoassays (LFIA) utilizing AuNPs produced via ablation techniques and monoclonal antibodies (MAB) hold immense potential. These AuNPs have already proven their worth in various applications, including diagnostics. The high demand for material is poised to transform the healthcare business, which potentially enables point-of-care devices that enhance healthcare accessibility. Therefore, improvements in reproducibility and scalability are crucial for mass production and widespread adoption in healthcare facilities. Alternatively, laser ablation of gold (Au) in aqueous medium has generated AuNPs in recent years. Intense laser light ablates an Au target immersed in aqueous fluids, resulting in Au nanoclusters. These laser-synthesized AuNPs are impurity-free because no extra chemicals were used, resulting in a high-purity particle without foreign ligands on the surface. Additionally, the absence of salt requirements in chemical processes for producing pure gold reduces manufacturing costs. However, because the reproducibility of this product is relatively low, another concern needs to be explored more.
